# 3-(2-Furylmethyl­ene)-1,5-dioxaspiro­[5.5]undecane-2,4-dione

**DOI:** 10.1107/S1600536809026877

**Published:** 2009-07-15

**Authors:** Wu-Lan Zeng, Fang-fang Jian

**Affiliations:** a MicroScale Science Institute, Department of Chemistry and Chemical Engineering, Weifang University, Weifang 261061, People’s Republic of China; b MicroScale Science Institute, Weifang University, Weifang 261061, People’s Republic of China

## Abstract

In the title mol­ecule, C_14_H_14_O_5_, the 1,3-dioxane ring is in an envelope conformation with the ring C atom common to the cyclo­hexane ring forming the flap. The other five atoms of the 1,3-dioxane ring are essentially planar [maximum deviation from the least-squares plane = 0.041 (3) Å] and form a dihedral angle of 13.75 (2)° with the furan ring. In the crystal structure, weak inter­molecular C—H⋯O hydrogen bonds form extended chains along [101].

## Related literature

For the applications and conformational features of spiro compounds, see: Yaozhong *et al.* (1998 ▶); Lian *et al.* (2008 ▶); Wei *et al.* (2008 ▶).
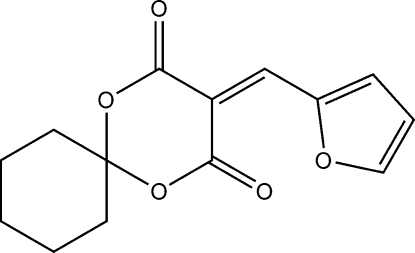


## Experimental

### 

#### Crystal data

C_14_H_14_O_5_
*M* *_r_* = 262.25
Triclinic, 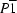

*a* = 7.0634 (14) Å
*b* = 9.5103 (19) Å
*c* = 10.183 (2) Å
α = 64.91 (3)°
β = 82.38 (3)°
γ = 84.76 (3)°
*V* = 613.6 (2) Å^3^
*Z* = 2
Mo *K*α radiation
μ = 0.11 mm^−1^
*T* = 293 K
0.18 × 0.15 × 0.12 mm

#### Data collection

Bruker SMART CCD diffractometer
Absorption correction: none
6043 measured reflections
2779 independent reflections
1456 reflections with *I* > 2σ(*I*)
*R*_int_ = 0.031

#### Refinement

*R*[*F* ^2^ > 2σ(*F* ^2^)] = 0.074
*wR*(*F* ^2^) = 0.285
*S* = 1.11
2779 reflections
172 parameters
H-atom parameters constrained
Δρ_max_ = 0.48 e Å^−3^
Δρ_min_ = −0.35 e Å^−3^

### 

Data collection: *SMART* (Bruker, 1997 ▶); cell refinement: *SAINT* (Bruker, 1997 ▶); data reduction: *SAINT*; program(s) used to solve structure: *SHELXS97* (Sheldrick, 2008 ▶); program(s) used to refine structure: *SHELXL97* (Sheldrick, 2008 ▶); molecular graphics: *SHELXTL* (Sheldrick, 2008 ▶); software used to prepare material for publication: *SHELXTL*.

## Supplementary Material

Crystal structure: contains datablocks global, I. DOI: 10.1107/S1600536809026877/lh2856sup1.cif

Structure factors: contains datablocks I. DOI: 10.1107/S1600536809026877/lh2856Isup2.hkl

Additional supplementary materials:  crystallographic information; 3D view; checkCIF report

## Figures and Tables

**Table 1:** Hydrogen-bond geometry (Å, °)

*D*—H⋯*A*	*D*—H	H⋯*A*	*D*⋯*A*	*D*—H⋯*A*
C2—H2*A*⋯O3^i^	0.97	2.59	3.470 (4)	151
C14—H14*A*⋯O3^ii^	0.93	2.50	3.230 (2)	135

Symmetry codes: (i) 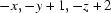; (ii) 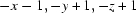.

**Fig. 1.**
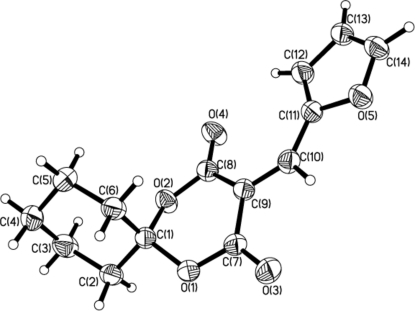
The molecular structure of (I), drawn with 30% probability ellipsoids and spheres of arbritrary size for the H atoms.

**Fig. 2.**
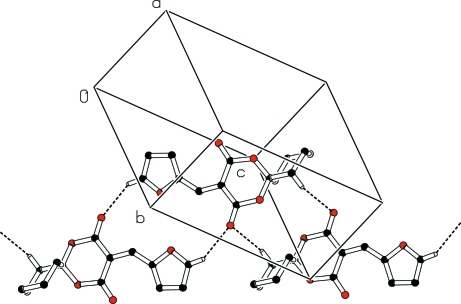
Part of the crystal structure of (I) with hydrogen bonds drawn as dashed lines.

## supplementary crystallographic information

### Comment

Spiro compounds are widely used in medicine, catalysis and optical material (Lian *et al.*, 2008; Yaozhong *et al.*, 1998; Wei *et al.*, 2008) owing to their interesting conformational features. We report here the synthesis and structure of the title compound, (I) (Fig. 1), as part of our ongoing studies on new spiro compounds with potentially higher bioactivity.

The 1,3-dioxane ring is in an envelope conformation with atom C1 forming the flap and the mean plane of the other five atoms (O1/O2/C1/C7—C9) form a dihedral angle of  13.75 (2)° with the furan ring O5/C11-C14). The crystal structure is stabilized by weak intermolecular C—H···O hydrogen bonds (Table 1, Fig. 2).

### Experimental

The mixture of malonic acid (6.24 g, 0.06 mol) and acetic anhydride(9 ml) in conc. sulfuric acid (0.25 ml) was stirred with water at 303K, After dissolving, cyclohexanone (5.88 g, 0.06 mol) was added dropwise into solution for 1 h. The reaction was allowed to proceed for 4 h. The mixture was cooled and filtered, and then an ethanol solution of furan-2-carbaldehyde (5.76 g,0.06 mol) was added. The solution was then filtered and concentrated. Single crystals were obtained by evaporation of an acetone-ethylacetate (2:1 *v*/*v*) solution of (I) at room temperature over a period of one week.

### Refinement

The H atoms were placed in calculated positions (C—H = 0.93–0.97 Å), and refined as riding with *U*_iso_(H) = 1.2*U*_eq_(C).

### Crystal data

**Table:**

C_14_H_14_O_5_	*Z* = 2
*M**_r_* = 262.25	*F*(000) = 276
Triclinic, *P*1	*D*_x_ = 1.420 Mg m^−^^3^
Hall symbol: -P 1	Mo *K*α radiation, λ = 0.71073 Å
*a* = 7.0634 (14) Å	Cell parameters from 6043 reflections
*b* = 9.5103 (19) Å	θ = 3.5–27.5°
*c* = 10.183 (2) Å	µ = 0.11 mm^−^^1^
α = 64.91 (3)°	*T* = 293 K
β = 82.38 (3)°	Block, colorless
γ = 84.76 (3)°	0.18 × 0.15 × 0.12 mm
*V* = 613.6 (2) Å^3^	

### Data collection

**Table:**

Bruker SMART CCD diffractometer	1456 reflections with *I* > 2σ(*I*)
Radiation source: fine-focus sealed tube	*R*_int_ = 0.031
graphite	θ_max_ = 27.5°, θ_min_ = 3.5°
ω scans	*h* = −9→9
6043 measured reflections	*k* = −12→12
2779 independent reflections	*l* = −13→13

### Refinement

**Table:**

Refinement on *F*^2^	Primary atom site location: structure-invariant direct methods
Least-squares matrix: full	Secondary atom site location: difference Fourier map
*R*[*F*^2^ > 2σ(*F*^2^)] = 0.074	Hydrogen site location: inferred from neighbouring sites
*wR*(*F*^2^) = 0.285	H-atom parameters constrained
*S* = 1.11	*w* = 1/[σ^2^(*F*_o_^2^) + (0.1559*P*)^2^ + 0.0401*P*] where *P* = (*F*_o_^2^ + 2*F*_c_^2^)/3
2779 reflections	(Δ/σ)_max_ < 0.001
172 parameters	Δρ_max_ = 0.48 e Å^−^^3^
0 restraints	Δρ_min_ = −0.35 e Å^−^^3^

### Special details

**Table:**

Geometry. All e.s.d.'s (except the e.s.d. in the dihedral angle between two l.s. planes) are estimated using the full covariance matrix. The cell e.s.d.'s are taken into account individually in the estimation of e.s.d.'s in distances, angles and torsion angles; correlations between e.s.d.'s in cell parameters are only used when they are defined by crystal symmetry. An approximate (isotropic) treatment of cell e.s.d.'s is used for estimating e.s.d.'s involving l.s. planes.
Refinement. Refinement of *F*^2^ against ALL reflections. The weighted *R*-factor *wR* and goodness of fit *S* are based on *F*^2^, conventional *R*-factors *R* are based on *F*, with *F* set to zero for negative *F*^2^. The threshold expression of *F*^2^ > σ(*F*^2^) is used only for calculating *R*-factors(gt) *etc*. and is not relevant to the choice of reflections for refinement. *R*-factors based on *F*^2^ are statistically about twice as large as those based on *F*, and *R*- factors based on ALL data will be even larger.

### Fractional atomic coordinates and isotropic or equivalent isotropic displacement parameters (Å^2^)

**Table:**

	*x*	*y*	*z*	*U*_iso_*/*U*_eq_	
O2	0.2535 (3)	0.2890 (3)	0.7823 (3)	0.0604 (7)	
O1	0.0683 (3)	0.4802 (3)	0.8363 (3)	0.0619 (7)	
O4	0.1675 (3)	0.1935 (4)	0.6368 (3)	0.0709 (8)	
O5	−0.4171 (3)	0.3169 (3)	0.4940 (3)	0.0651 (7)	
O3	−0.2004 (4)	0.5790 (3)	0.7400 (3)	0.0726 (8)	
C9	−0.0519 (5)	0.3713 (4)	0.6897 (4)	0.0539 (8)	
C2	0.3594 (5)	0.3844 (4)	0.9383 (4)	0.0611 (9)	
H2A	0.3191	0.4343	1.0035	0.073*	
H2B	0.4318	0.4577	0.8525	0.073*	
C7	−0.0735 (5)	0.4822 (4)	0.7561 (4)	0.0550 (8)	
C8	0.1248 (4)	0.2756 (4)	0.7003 (4)	0.0524 (8)	
C10	−0.2000 (5)	0.3676 (4)	0.6195 (4)	0.0556 (9)	
H10A	−0.3012	0.4351	0.6257	0.067*	
C1	0.1860 (5)	0.3406 (4)	0.8941 (4)	0.0553 (8)	
C13	−0.2757 (5)	0.1442 (5)	0.4174 (4)	0.0637 (9)	
H13A	−0.2541	0.0765	0.3723	0.076*	
C6	0.0755 (5)	0.2159 (4)	1.0210 (4)	0.0591 (9)	
H6A	−0.0295	0.1863	0.9867	0.071*	
H6B	0.0224	0.2560	1.0918	0.071*	
C11	−0.2361 (4)	0.2852 (4)	0.5395 (4)	0.0542 (8)	
C14	−0.4363 (6)	0.2284 (5)	0.4246 (5)	0.0697 (10)	
H14A	−0.5473	0.2254	0.3860	0.084*	
C5	0.2062 (6)	0.0738 (5)	1.0931 (5)	0.0708 (11)	
H5A	0.1356	−0.0024	1.1779	0.085*	
H5B	0.2478	0.0272	1.0254	0.085*	
C4	0.3771 (6)	0.1165 (5)	1.1386 (5)	0.0770 (12)	
H4A	0.3361	0.1520	1.2146	0.092*	
H4B	0.4608	0.0251	1.1787	0.092*	
C3	0.4854 (5)	0.2409 (5)	1.0140 (5)	0.0751 (12)	
H3A	0.5407	0.2005	0.9440	0.090*	
H3B	0.5893	0.2702	1.0496	0.090*	
C12	−0.1479 (5)	0.1787 (5)	0.4913 (4)	0.0654 (10)	
H12A	−0.0241	0.1369	0.5055	0.078*	

### Atomic displacement parameters (Å^2^)

**Table:**

	*U*^11^	*U*^22^	*U*^33^	*U*^12^	*U*^13^	*U*^23^
O2	0.0605 (14)	0.0740 (17)	0.0588 (14)	0.0104 (11)	−0.0124 (11)	−0.0400 (13)
O1	0.0783 (16)	0.0523 (15)	0.0644 (15)	0.0134 (11)	−0.0225 (12)	−0.0321 (12)
O4	0.0637 (14)	0.089 (2)	0.0877 (19)	0.0176 (13)	−0.0172 (13)	−0.0652 (17)
O5	0.0641 (14)	0.0686 (17)	0.0778 (17)	0.0096 (11)	−0.0223 (12)	−0.0433 (14)
O3	0.0799 (17)	0.0654 (17)	0.0807 (18)	0.0260 (13)	−0.0212 (14)	−0.0403 (15)
C9	0.0649 (19)	0.0522 (19)	0.0511 (18)	0.0083 (14)	−0.0129 (14)	−0.0280 (16)
C2	0.065 (2)	0.060 (2)	0.067 (2)	−0.0029 (16)	−0.0113 (16)	−0.0333 (18)
C7	0.0639 (19)	0.0490 (19)	0.0562 (19)	0.0063 (14)	−0.0102 (15)	−0.0264 (16)
C8	0.0567 (18)	0.056 (2)	0.0497 (17)	0.0035 (14)	−0.0042 (13)	−0.0288 (16)
C10	0.0621 (19)	0.055 (2)	0.0515 (18)	0.0086 (14)	−0.0084 (14)	−0.0254 (16)
C1	0.070 (2)	0.052 (2)	0.0520 (18)	0.0160 (15)	−0.0160 (15)	−0.0307 (16)
C13	0.079 (2)	0.060 (2)	0.067 (2)	0.0098 (17)	−0.0219 (17)	−0.0397 (19)
C6	0.0599 (19)	0.059 (2)	0.066 (2)	−0.0009 (15)	−0.0098 (16)	−0.0322 (18)
C11	0.0547 (18)	0.0522 (19)	0.061 (2)	0.0055 (13)	−0.0150 (14)	−0.0279 (17)
C14	0.074 (2)	0.072 (3)	0.084 (3)	0.0043 (18)	−0.0272 (19)	−0.049 (2)
C5	0.089 (3)	0.053 (2)	0.066 (2)	0.0002 (18)	−0.0142 (19)	−0.0202 (19)
C4	0.093 (3)	0.066 (3)	0.077 (3)	0.018 (2)	−0.036 (2)	−0.031 (2)
C3	0.063 (2)	0.079 (3)	0.101 (3)	0.0110 (19)	−0.028 (2)	−0.052 (3)
C12	0.067 (2)	0.065 (2)	0.077 (2)	0.0113 (17)	−0.0228 (18)	−0.041 (2)

### Geometric parameters (Å, °)

**Table:**

O2—C8	1.361 (4)	C13—C14	1.340 (5)
O2—C1	1.433 (4)	C13—C12	1.390 (5)
O1—C7	1.368 (4)	C13—H13A	0.9300
O1—C1	1.438 (4)	C6—C5	1.525 (5)
O4—C8	1.206 (4)	C6—H6A	0.9700
O5—C14	1.333 (5)	C6—H6B	0.9700
O5—C11	1.382 (4)	C11—C12	1.371 (5)
O3—C7	1.199 (4)	C14—H14A	0.9300
C9—C10	1.354 (5)	C5—C4	1.495 (6)
C9—C8	1.463 (5)	C5—H5A	0.9700
C9—C7	1.463 (5)	C5—H5B	0.9700
C2—C1	1.507 (5)	C4—C3	1.493 (6)
C2—C3	1.522 (6)	C4—H4A	0.9700
C2—H2A	0.9700	C4—H4B	0.9700
C2—H2B	0.9700	C3—H3A	0.9700
C10—C11	1.405 (5)	C3—H3B	0.9700
C10—H10A	0.9300	C12—H12A	0.9300
C1—C6	1.512 (5)		
			
C8—O2—C1	118.6 (2)	C5—C6—H6A	109.6
C7—O1—C1	117.9 (3)	C1—C6—H6B	109.6
C14—O5—C11	107.2 (3)	C5—C6—H6B	109.6
C10—C9—C8	124.7 (3)	H6A—C6—H6B	108.1
C10—C9—C7	116.0 (3)	C12—C11—O5	107.1 (3)
C8—C9—C7	119.3 (3)	C12—C11—C10	140.0 (3)
C1—C2—C3	110.5 (3)	O5—C11—C10	112.9 (3)
C1—C2—H2A	109.5	O5—C14—C13	111.5 (3)
C3—C2—H2A	109.5	O5—C14—H14A	124.2
C1—C2—H2B	109.5	C13—C14—H14A	124.2
C3—C2—H2B	109.5	C4—C5—C6	111.3 (3)
H2A—C2—H2B	108.1	C4—C5—H5A	109.4
O3—C7—O1	117.7 (3)	C6—C5—H5A	109.4
O3—C7—C9	125.2 (3)	C4—C5—H5B	109.4
O1—C7—C9	117.0 (3)	C6—C5—H5B	109.4
O4—C8—O2	118.5 (3)	H5A—C5—H5B	108.0
O4—C8—C9	125.1 (3)	C3—C4—C5	111.9 (3)
O2—C8—C9	116.3 (3)	C3—C4—H4A	109.2
C9—C10—C11	134.7 (3)	C5—C4—H4A	109.2
C9—C10—H10A	112.7	C3—C4—H4B	109.2
C11—C10—H10A	112.7	C5—C4—H4B	109.2
O2—C1—O1	110.2 (3)	H4A—C4—H4B	107.9
O2—C1—C2	106.7 (3)	C4—C3—C2	112.3 (3)
O1—C1—C2	106.3 (3)	C4—C3—H3A	109.1
O2—C1—C6	111.0 (3)	C2—C3—H3A	109.1
O1—C1—C6	110.2 (3)	C4—C3—H3B	109.1
C2—C1—C6	112.3 (3)	C2—C3—H3B	109.1
C14—C13—C12	106.0 (4)	H3A—C3—H3B	107.9
C14—C13—H13A	127.0	C11—C12—C13	108.1 (3)
C12—C13—H13A	127.0	C11—C12—H12A	125.9
C1—C6—C5	110.3 (3)	C13—C12—H12A	125.9
C1—C6—H6A	109.6		

### Hydrogen-bond geometry (Å, °)

**Table:**

*D*—H···*A*	*D*—H	H···*A*	*D*···*A*	*D*—H···*A*
C2—H2A···O3^i^	0.97	2.59	3.470 (4)	151
C14—H14A···O3^ii^	0.93	2.50	3.230 (2)	135
C10—H10A···O3	0.93	2.34	2.764 (3)	107
C12—H12A···O4	0.93	2.27	2.879 (2)	122

Symmetry codes: (i) −*x*, −*y*+1, −*z*+2; (ii) −*x*−1, −*y*+1, −*z*+1.
